# Systemic IL-10 and IFN-γ Levels in Respiratory Syncytial Virus- and Rhinovirus-Infected Bulgarian Children with Acute Bronchiolitis and Their Impact on Clinical Manifestation

**DOI:** 10.3390/pathogens14050426

**Published:** 2025-04-27

**Authors:** Emiliya Kostadinova, Svetla Angelova, Tsvetana Tsonkova-Popova, Dima Zlateva, Rozalina Yordanova, Spaska Stanilova

**Affiliations:** 1 Department of Pediatrics, Medical Faculty, Trakia University, 6000 Stara Zagora, Bulgaria; emiliya.kostadinova@trakia-uni.bg (E.K.); tsvetana.tsonkova@trakia-uni.bg (T.T.-P.); dimazlateva@yahoo.com (D.Z.); 2 Department of Hygiene, Epidemiology, Microbiology, Parasitology and Infectious Diseases, Medical Faculty, Trakia University, 6000 Stara Zagora, Bulgaria; svetla.angelova@trakia-uni.bg; 3 Department of Health Care, Medical College, Trakia University, 6000 Stara Zagora, Bulgaria; rozalina.yordanova@trakia-uni.bg; 4 Department of Molecular Biology, Immunology and Medical Genetics, Medical Faculty, Trakia University, 6000 Stara Zagora, Bulgaria

**Keywords:** serum IL-10, RSV, RV, bronchiolitis, serum IFN-γ

## Abstract

Respiratory syncytial virus (RSV) and rhinovirus (RV)—the two primary causative viruses of bronchiolitis in children—have been studied extensively in terms of their associations with disease severity and serious late disease outcomes. In this study, we explored the differences in the clinical values of IFN-γ and IL-10 serum levels in RSV and RV bronchiolitis in the Bulgarian childhood population. Eighty-eight children with acute bronchiolitis, aged two months to two years, who were admitted to the General Pediatrics Clinic of University Hospital “Prof. St. Kirkovich”, Stara Zagora, Bulgaria served as this study’s subjects. The degree of wheezing and respiratory failure were classified. Naso-pharyngeal swabs were collected from all participants, and molecular identification of viruses was performed using real-time PCR. Serum samples were used to determine IFN-γ and IL-10 quantities using ELISA kits, and data are presented as the median IQR (25–75%). The total serum IL-10 levels were significantly enhanced in RSV-infected children compared to those infected with RV (14.4 (12.2–24.0) vs. 8.9 (7.2–12.5); *p* < 0.001) and the other viral bronchiolitis groups (14.4 (12.2–24.0) vs. 6.65 (3.9–15.3); *p* = 0.003). The highest IL-10 levels (14.4 (12.8–27.9)) were found in RSV-positive patients with first-degree respiratory failure. Almost identical serum IFN-γ levels were determined for RSV- and RV-positive bronchiolitis patients (3.2 (1.6–6.8) and 2.8 (1.1–7.3); *p* = 0.781). Variance analysis of IL-10 serum levels revealed statistically significant differences among the patient groups depending on the type of viral infection, concerning respiratory failure (*p* = 0.005) and wheezing severity (*p* = 0.017). Our findings show that the IL-10 levels and the type of virus have a combined effect on disease severity. These data might contribute to patients’ personalized/individualized therapy and the prevention of recurrent wheezing later in life.

## 1. Introduction

Acute lower respiratory tract infections in children under two years of age are a significant health burden. The common clinical manifestation of respiratory disorders is acute viral bronchiolitis, associated with increased morbidity and treatment costs, as reported in previous narrative reviews. The American Academy of Pediatrics (AAP) defines bronchiolitis as a constellation of symptoms, including viral prodromal upper respiratory tract manifestations (rhinorrhea, fever, and cough) followed by difficulty of breathing and bronchial obstruction (wheezing) in children younger than two years of age [1]. Although the course is uncomplicated in mild forms, bronchiolitis is the most common infection of the terminal airways and a major cause of hospitalization in children under two years of age, often presenting as acute respiratory failure (ARF) and requiring hospitalization in approximately 3% of cases and intensive care in 2–6% [2].

Recently developed PCR-based assays have made it possible to identify various viruses that can cause bronchiolitis. The reported viruses mainly include respiratory syncytial virus (RSV) and rhinoviruses (RVs), although parainfluenza viruses (PIVs), metapneumoviruses (HMPVs), coronaviruses (HCOVs), adenoviruses (AdVs), influenza viruses (IVs), human bocavirus, and enterovirus have also been identified [1,2,3]. Real-time PCR diagnostics have reached a 100% virus detection rate in severe bronchiolitis [4].

Despite many viruses, studies have shown that RSV is the most common pathogen, causing 50 to 80% of all hospitalizations for acute viral bronchiolitis [5,6,7]. A retrospective study of 274 children under two years of age admitted to pediatric intensive care with bronchiolitis over a six-year period (2011–2016) reported that 60% were infected with RSV, followed by 26% infected with rhinovirus [8]. The authors also concluded that RSV causes more severe bronchiolitis than other viruses, although some previous reports have stated that the clinical severity of viral bronchiolitis does not depend on the type of virus [9,10].

Studies using traditional viral techniques and conventional PCR in four regions of Bulgaria found a significant prevalence (39%) of RSV as the causative agent of bronchiolitis in hospitalized children under one year of age [11]. Our previous clinicoepidemiological studies of hospitalized patients with acute bronchiolitis under two years of age confirmed the leading role of RSV infection, found in 50% of all etiologically proven bronchiolitis, with a male predominance and higher prevalence among urban children. Our results also indicated that RSV is the most frequent etiologic agent in winter and spring (65.3%), whereas in summer, RV is the leading etiologic agent, found in 40% of cases [12].

Over the past decade, the heterogeneity of viral bronchiolitis has been recognized based on the etiology, host immune response, and pathophysiological mechanisms that affect the clinical presentation of three major types: RSV-induced bronchiolitis, RV-induced bronchiolitis, and wheezing due to other, less frequent and less severe, viral infections [13,14].

Most RSV- and RV-infected children develop mild diseases. However, in some infants, this infection can lead to severe clinical presentation and further chronic complications. Overall, the immune responses elicited by RSV and RV can be both protective and pathogenic and vary between individuals due to the complex interplay between genetic and environmental factors. Both viral genome and host characteristics, such as age, a maturing immune system, personal and family history of atopy, and comorbidities, play crucial roles in the clinical severity of bronchiolitis and its long-term outcomes [14,15,16].

RSV and RV are known to cause significant damage to the lung epithelium because of the excessive inflammatory response in the airways. Cytokines are essential regulatory proteins of the immune response, involved in the modulation of the inflammatory process and the development of a protective or damaging immune response. The amount and combination of cytokines secreted under conditions of an induced immune response are key to the realization of normal or pathogenic immune responses in the body [17]. In recent years, cytokine models have been studied in the search for potential cytokine biomarkers associated with the severity and prognosis of RSV-related lower airway inflammation. Interferon-alpha (IFN-alpha) and IL-6, as well as Th2-type cytokines, have been studied for their association with the severity of RSV bronchiolitis [18].

The T helper 1/T helper 2 axis and T regulatory cells of the adaptive immune response and their associated cytokine imbalance play pivotal roles in the immune-mediated pathology of RSV and RV bronchiolitis [19,20,21]. A few studies have demonstrated an association between the main Th1/Th2-regulated cytokines, particularly IFN-γ and IL-10, with some clinical features of RSV or RV bronchiolitis [22,23]. Analyzing Th1 cytokines, such as IFN-γ and TNF-a, and Th2 cytokines, such as IL-4, IL-6, and IL-10, presents a challenge in elucidating the immune mechanisms in lung tissue during acute viral bronchiolitis. To the best of our knowledge, the role of cytokines in viral bronchiolitis in children under two years of age from the Bulgarian population has not yet been well studied. Nevertheless, the differences between these two viruses and the serum levels of IFN-γ and IL-10 concerning clinical severity and long-term outcomes are not fully understood.

The purpose of this study was to evaluate the association between the serum levels of IFN-γ and IL-10 and the clinical features of RSV and RV bronchiolitis in the Bulgarian childhood population.

## 2. Materials and Methods

### 2.1. Study Subjects

The subjects of this prospective study were 88 children aged 2 months to 2 years, hospitalized from September 2021 to March 2023 in the General Pediatric Clinic of University Hospital “Prof. St. Kirkovitch” Stara Zagora, Bulgaria, with clinical manifestations of acute bronchiolitis. The clinical course was presented according to the etiologic agent, the degree of bronchial obstruction (wheezing), and clinical and laboratory evidence of acute respiratory failure as markers of severity.

The criteria for hospitalized children with bronchiolitis included an impaired general condition, impaired feeding, manifestations of dehydration, tachypnea/apnea, expiratory-type dyspnea, wheezing, retraction, thoraco-abdominal asynchronism, nasal breathing, cyanosis, muscle hypotony, pulmonary auscultatory findings of fine crepitations, and radiographic evidence of pulmonary hyperventilation. The criteria for wheezing were the presence of a cough, wheezing with expiratory-type dyspnea, cyanosis, retraction, and abnormality of blood gas analysis (BGA). Depending on the severity, wheezing was defined as mild, moderate, or severe: mild (wheezing on auscultation; no breathlessness or cyanosis at rest; normal range of BGA indices; the child’s state of health, as a rule, is not suffering); moderate (wheezing heard at a distance; expiratory or mixed dyspnea at rest; perioral cyanosis; retraction of the chest musculature; BGA indices are mildly impaired (pO_2_ > 60 mm Hg; pCO_2_ < 45 mm Hg)); severe (state of health of the child is suffering; noisy shortness of breath with auxiliary muscle participation; cyanosis; reduced BGA indices (pO_2_ < 60 mm Hg; pCO_2_ > 45 mm Hg)) [24].

The criteria for acute respiratory failure (ARF) were clinical findings of tachypnea (according to age) manifested on exertion or at rest and laboratory findings of BGA: first (I) grade (PaO_2_ 50–60 mm Hg; SatO_2_ 92–94%; PaCO_2_ ≥ 45 mm Hg), second (II) grade (PaO_2_ 40–50 mm Hg; SatO_2_ ≤ 92%; PaCO_2_ ≥ 45 mm Hg; +/−acidosis), and third (III) grade (PaO_2_ < 40 mm Hg; SatO_2_ ≤ 90%; PaCO_2_ ≥ 45 mm Hg; +/−acidosis) [24].

Routine blood tests (a complete blood count (CBC), differential formula of white blood cells (WBC), and CRP), basic biochemical parameters, BGA, and RT-PCR examination of nasopharyngeal aspirate/throat swabs were performed in all patients. Bacterial infection was ruled out using conventional clinical and laboratory indices. The clinical follow-up spanned a 2-year period and included the processing of questionnaires completed by parents and general practitioners for monitoring clinical status or clinical control when hospitalization occurred, which was reported as a recurrence. As a recurrence, we noted at least one subsequent hospitalization of the patient with bronchiolitis in the follow-up period.

Demographic characteristics and some of the clinical manifestations stratified by viral infection are presented in Table 1.

### 2.2. Nucleic Acid Extraction and Real-Time PCR Assay

Nasopharyngeal swabs were collected from 88 hospitalized children with bronchiolitis in an appropriate viral transport medium, satisfying the requirements of RT-PCR analysis. The swabs were stored at 4 °C for up to 24 h before being sent and were processed immediately in the laboratory or stored at −80 °C. Viral DNA and RNA were extracted by an automatic extraction Insta NX^®^ system (Himedia, Maharashtra, India) using an Insta NX™ Viral DNA/RNA Purification Kit in accordance with the manufacturer’s instructions. SARS-CoV-2, influenza viruses A/B, and RSV were detected using real-time RT-PCR employing a 1copy™ COVID-19/FluA/FluB/RSV qPCR Kit (Clinomics Inc., Seongnam-si Korea). Primers, probes, and positive controls for detection/typing of the SARS-CoV-2, influenza A/B, and RSV viruses are part of the commercial 1copy™ qPCR reagent. This kit specifically identifies two regions in the SARS-CoV-2 genome: N-gene and RdRp-gene. The detection limits were 400 copies/mL for SARS-CoV-2 and 800 copies/mL for FluA/B and RSV. Screening for human metapneumovirus (HMPV), parainfluenza virus (PIV) types 1/3, rhinovirus (RV), adenovirus (AdV), and four seasonal human coronaviruses (OC43, 229E, NL63, and HKU1) was performed using singleplex real-time RT-PCR assays with an AgPath-IDTM One-Step RT-PCR Kit (Thermo Fisher Scientific, Carlsbad, CA, USA). The primer sequences, Taqman^®^ probes (FAM/BHQ), and thermocycling conditions for real-time PCR assays were identical to those previously presented [12,25]. A cycle threshold (Ct) value < 38 was considered positive. Amplification of all the target viruses was performed using the 96-well CFX96TM real-time PCR detection system (Bio-Rad Laboratories, Inc., Hercules, CA, USA).

Since this study did not thoroughly test all of the non-influenza viruses that cause bronchiolitis, particularly bocavirus and some other viruses, the group of patients without a confirmed pathogen was referred to as a “group with undetected viruses”.

### 2.3. Quantification of Serum IL-10 and IFN-γ Concentrations

Upon admission of each patient, before the start of treatment, venous blood was taken, and the serum was separated by centrifugation. The separated serum was stored at −70 °C until testing. Serum IL-10 and IFN-γ levels were measured using enzyme-linked immunosorbent assay (ELISA) kits (Diaclone, Besancon, France) according to the manufacturer’s instructions. A standard curve constructed with the kit’s standards was used to determine the cytokine concentrations expressed in picograms per milliliter (pg/mL). Patient serum samples were run in duplicates and analyzed in the same analytic batch. The minimum detectable level of IL-10 was less than 0.98 pg/mL for the Diaclone Human IL-10 High Sensitivity ELISA Kit. The sensitivity or minimum detectable dose of IFN-γ using the Diaclone Human IFN-γ High Sensitivity ELISA Kit was 0.69 pg/mL.

### 2.4. Ethical Approval

The study protocol was approved by the ethics committee of the Medical Faculty, Trakia University, Stara Zagora, Bulgaria as a part of Research Grant № 2/2021. This study was conducted in accordance with the principles of the Helsinki Declaration and good clinical practices. Written informed consent was obtained from the parents or legal guardians of all studied children after a detailed explanation of this study. All participants were Caucasian.

### 2.5. Statistical Analysis

All data for this study were analyzed using SPSS Statistics, version 29.0, for Windows (SPSS Inc., Chicago, IL, USA). The studied cytokine concentrations were non-normally distributed. Cytokine concentration data are presented as the median and interquartile range (IQR) (25–75%). ANOVA and MANOVA were used to assess the relationships between the serum IL-10 levels and the studied types of viruses and/or clinical manifestations. When appropriate, the cytokine concentrations between RSV- and RV-infected patients were compared using the Kruskal–Wallis or Mann–Whitney U-test. Pearson’s chi-square (χ^2^) test and correlational analyses were also used in relevant cases for categorical variables. A *p*-value < 0.05 was considered statistically significant.

## 3. Results

### 3.1. Serum IL-10 and IFN-γ Levels in Bronchiolitis Children in Relation to the Type of Infecting Virus

**Figure 1:**
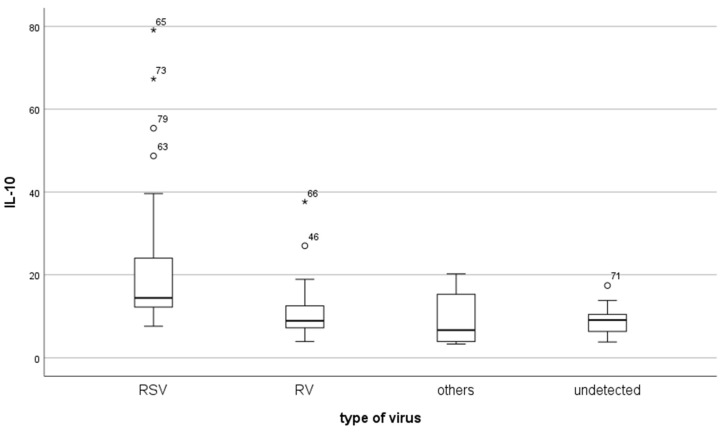
Serum concentrations of IL-10 (pg/mL) in groups of bronchiolitis children infected with RSV, RV, other viruses, and undetected viruses. The results are presented as the median with interquartile range (IQR 25–75%) and non-outlier range (whiskers). Outliers are marked with circles, and extremes are marked with asterisks. Statistical significances were calculated using the Mann–Whitney test for RSV-positive patients against groups of RV-positive (*p* < 0.001) and other virus-infected children (*p* = 0.003).

The total serum IL-10 levels (median IQR (25–75%)) in the studied groups were highest in the RSV-infected children (14.4 (12.2–24.0)), followed by those infected with RV (8.9 (7.2–12.5)). The serum IL-10 levels in the group of children infected with another virus were slightly lower than those in the group with an undetected type of virus infection. The group of patients infected with another virus included children infected with HMPV, AdV, SARS-CoV-2, and influenza viruses A/B. The results are presented in Table 2 and Figure 1. Statistical significances were calculated for the RSV-positive patients against the RV-positive (*p* < 0.001) and other virus-infected children (*p* = 0.003). The total IL-10 mean in the group of virus-infected children was also significantly higher than that of the group of children with an undetected infection: 12.6 (8.4–18.3) vs. 9.1 (6.4–10.5) (*p* < 0.001).

**Figure 2:**
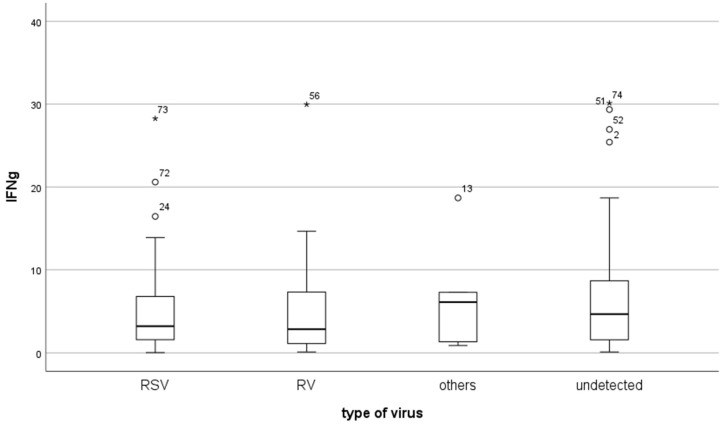
Serum concentrations of IFN-γ (pg/mL) in bronchiolitis children infected with RSV, RV, other viruses, and undetected viruses. The results are presented as the median and interquartile range (IQR 25–75%) and non-outlier range (whiskers). Outliers results are marked with circles, and extremes are marked with asterisks. No statistical significance between groups was calculated and analyzed using the Mann–Whitney test.

The total IFN-γ levels were the highest in the group of children with “other” viral infections (6.1 (1.34–7.28)), but the differences did not reach statistical significance against the other groups. The results are presented in Table 1 and Figure 2. Almost identical serum IFN-γ levels were observed in the RSV- and RV-positive patients (3.21 (1.58–6.78) and 2.85 (1.12–7.32); *p* = 0.781). Insignificantly increased IL-10 levels were calculated for children infected with another virus (6.1 (1.34–7.28)). The mean total IFN-γ levels in the group of virus-infected children were non-significantly decreased compared to the group of infected children with undetected viruses (3.1 (1.4–7.3) vs. 4.7(1.6–8.7); *p* = 0.335)).

### 3.2. Association Between IL-10 and IFN-γ Serum Levels in Children with Bronchiolitis in Relation to Respiratory Failure and Wheezing Severity

Variance analysis (ANOVA) of IL-10 serum levels revealed statistically significant differences in patient groups depending on the type of viral infection, as well as in relation to patient respiratory failure (*p* = 0.005) and wheezing severity (*p* = 0.017), as demonstrated in Table 3. Other clinical manifestations and demographic characteristics included in this study (Table 1) did not demonstrate any statistically significant differences. The patients infected with RSV and RV manifested first- and second-degree respiratory failure, whereas the absence of respiratory failure was detected in the bronchiolitis patients infected with viruses other than those studied. The highest IL-10 levels were found in the RSV-infected patients with first-degree respiratory failure. When comparing first- and second-degree respiratory failure in the RSV- and RV-infected patients, we found that RSV infection was associated with a threefold risk of developing second-degree respiratory failure (χ^2^ = 3.845; OR = 3.33; *p* = 0.050). Regardless of the viral type, high IL-10 levels were associated with bronchiolitis recurrence (*p* = 0.021; Mann–Whitney test).

Changes in IFN-γ serum levels between groups divided by viral infection did not correlate with the degree of respiratory failure (*p* = 0.441). We found the opposite tendency for IFN-γ levels in the RSV- and RV-positive patients, but the differences were not significant. When comparing first- and second-degree respiratory failure in the RSV-positive children vs. those with undetected viruses, we calculated a borderline statistically significant difference (*p* = 0.052, Mann–Whitney test). Generally, the mean IFN-γ levels across the different virus-infected groups showed a similar trend with wheezing severity as observed with respiratory failure. The children infected with RSV with a moderate severity of wheezing had the highest IL-10 levels, reaching statistical significance compared to those with a mild severity (14.4 (12.6–27.9) vs. 12.8 (12.4–15.6); *p* = 0.021 by Mann–Whitney test).

When comparing mild and severe degrees of wheezing severity in the RSV- and RV-infected patients (using crosstabulation), no significant predisposition was found (χ^2^ = 0.305; *p* = 0.581). The results showed that both viruses had approximately the same effect on the degree of wheezing severity. However, separately, the RSV or RV virus-infected children showed significant differences compared to the group of children with undetected viruses (χ^2^ = 9.118; *p* = 0.003 for RSV and χ^2^ = 8.561; *p* = 0.003 for RV).

### 3.3. Correlation of Serum IL-10 and IFN-γ Levels According to the Type of Viral Infection and Clinical Characteristics in Bulgarian Children with Bronchiolitis

The correlation results, as determined by Spearman’s rho coefficient, are presented in Table 4. A strong significant correlation was found between the detected viral infection and IL-10 (r = 0.546; *p* < 0.001). A weak and significant correlation was observed between viral species and wheezing severity (r = 0.219; *p* = 0.041). As expected, a strong and significant correlation between respiratory failure and wheezing severity was also calculated (r = 0.523; *p* < 0.001). Furthermore, recurrence was significantly correlated with wheezing.

## 4. Discussion

RSV and RV, the two primary viruses that cause bronchiolitis in children, have been extensively studied in terms of their association with disease severity and serious disease outcomes. In this study, we explored the differences in the clinical value of IFN-γ and IL-10 serum levels in RSV and RV bronchiolitis in the Bulgarian pediatric population.

We found a significantly higher level of IL-10 in the RSV-positive group of patients than in the RV-positive group and the other groups with viral bronchiolitis. The highest quantity of IL-10 was found in the RSV-infected patients with first-degree respiratory failure and moderate wheezing. In contrast, the serum IFN-γ levels did not differ significantly between the studied groups. Similar to our findings, other authors have not reported significant differences in serum IFN-γ concentrations between patients with RSV and RV bronchiolitis [26,27].

IL-10 is a major immunosuppressive cytokine produced by many cells involved in both innate and adaptive immunity. The early production of IL-10 by immune cells regulates RSV-elicited inflammation and prevents excessive and unresolved inflammatory adverse effects. Studies have shown that most of the airway damage caused by RSV is mediated by a pathogenic immune response and is not caused directly by the virus [28]. Along with this early complication, the RSV-induced Th2-shifted immune response has been linked with the development of exacerbated allergic responses later in life. Between 16% and 48% of infants hospitalized for RSV-induced bronchiolitis develop childhood asthma [29].

Experimental RSV infection of IL-10-deficient mice has been shown to result in more severe disease, greater pronounced neutrophilia infiltration in the lung tissue, increased levels of pro-inflammatory cytokines, and delayed recovery compared to wild mice [30,31]. The authors also reported that the peak of IL-10 production corresponded with the peak of developing virus-specific CD4+ T cell expansion, independent of the IFN-γ production.

Recently, it has become widely accepted that IL-10 plays a crucial role in acute RSV and RV infection in children, as well as in the recovery phase, although the reported data are conflicting. Some authors have associated increased serum IL-10 levels with disease severity. Alonso et al. reported that plasma IL-10 levels were higher in RSV-infected children with significant hypoxia compared to those without hypoxia [32]. Leahy et al. also found that increased serum IL-10 levels distinguished moderate from severe disease [20]. This finding is in line with the data reported by Hoebee et al., showing that single-nucleotide polymorphisms located in the promoter region of the IL10 gene are associated with increased resistance to severe RSV infection in children [33]. However, another study has reported no significant changes in IL-10 levels in RSV bronchiolitis and a lack of association with disease severity [34].

These contradictory results may be due to the complex regulatory role of IL-10 depending on the developed anti-RSV immune response stage. Most of the data showed the benefit of increased IL-10 in the acute phase of RSV bronchiolitis, but in later phases, including the convalescent phase, the effect might be adverse. Previously, a correlation was observed between increased ex vivo production of IL-10 from non-specifically stimulated monocytes during the convalescent phase of RSV infection and recurrent wheezing in infants during the year following RSV bronchiolitis [35]. Similarly, increased IL-10 levels in undiluted nasopharyngeal aspirates have been associated with physician-diagnosed post-bronchiolitis wheezing one year after infection [36]. The dual role of IL-10 depends on when this cytokine is elevated in RSV-induced lung inflammation, which has been demonstrated in a transgenic mouse model capable of overproducing IL-10 in the lungs. The authors reported that elevated IL-10 in the early phase of RSV infection not only inhibits acute inflammation and significantly alters lung immunopathology but also modulates the late immune response toward the Th2 type [37]. New evidence has shown that IL-10 and IL-1β can reverse the effects of RSV-infected CD16+ monocytes on CD4+ Treg proliferation [38]. Monitoring IL-10 in hospitalized children with acute bronchiolitis may help differentiate the clinical phenotype and implement a specific therapeutic strategy to prevent further development of wheezing and asthmatic responses in infants. Furthermore, future RSV vaccines must induce a balanced CD4 T cell response to facilitate viral clearance while inducing proper regulation of the immune response.

Our results showed a significant difference in the serum IL-10 levels between RSV and RV bronchiolitis patients. Similar findings were reported by Lee et al. [23].

The disease severity of bronchiolitis depends on the degree of respiratory failure and wheezing. We observed the effect of viral types on these clinical manifestations (presentation). We found significantly increased respiratory failure in RSV bronchiolitis patients compared to RV bronchiolitis patients and no significant impact on wheezing severity, which is in line with the results reported by Ding et al. [39].

Although elevated IL-10 levels were detected in RSV-infected patients with first-degree respiratory failure and moderate wheezing compared to the same clinical features in RV-infected patients, the correlation analysis revealed that these differences might be more related to the viral types rather than the IL-10 levels (Table 4). Our findings showed that both the IL-10 levels and the virus type have a combined effect on disease severity. Different viruses can elicit the characteristic immune profile in bronchiolitis. Hurme et al. demonstrated different cytokine profiles between RSV- and RV-infected children and their impact on disease severity [40].

A limitation of our study is the small number of some studied groups, and further studies should be performed to confirm the role of IL-10 depending on viral type.

Differences in disease severity cannot be excluded, as they depend on individual host susceptibility to RSV, RV, or other viruses. Supporting this, Hurme et al. reported similar findings when comparing inpatient and outpatient groups with RV-induced bronchiolitis [41]. Many single-nucleotide polymorphisms in immune response genes have been associated with severe bronchiolitis [42,43]. Moreover, some of these SNPs are implicated in abnormal Th2 polarization of the immune response during RSV infection and childhood asthma development [29,44].

Taken together, all these data highlight the possibility of differentiating bronchiolitis endotypes depending on the virus type and signature cytokine profiles determined by the host genome, including IL-10. Understanding these mechanisms might contribute not only to better personalized/individualized treatment options for patients but also to preventing the development of long-term adverse effects on the lungs like childhood asthma.

## 5. Conclusions

RSV and RV infection—the two primary causes of bronchiolitis in early childhood—have been studied extensively in terms of their association with disease severity and serious late disease outcomes. We found significantly higher IL-10 levels in the RSV-positive group of patients compared to the RV-positive groups and the other groups with viral bronchiolitis. The highest IL-10 levels were found in RSV-infected patients with first-degree respiratory failure. In contrast, serum IFN-γ levels did not differ significantly between the studied groups. Additionally, we found significantly increased respiratory failure in patients with RSV bronchiolitis compared to those with RV bronchiolitis and no significant impact on wheezing severity. Our data provide evidence of the substantial role of IL-10 in differentiating endotypes of bronchiolitis, potentially contributing to personalized treatment.

## Figures and Tables

**Table 1:** Demographic characteristics and clinical data of Bulgarian children with bronchiolitis.

	*RSV*	*RV*	*Others*	*Undetected Virus*	*Intergroup*
	n = 36	n = 22	n = 6	n = 24	Significance
*Sex*					
*Male*	22	13	4	13	*p* = 0.935
*female*	14	9	2	11	
*Age (months)*	8.94	12.32	9	11.79	*p* = 0.313
*Respiratory failure*					
*No*	0	0	0	8	RSV vs. RV
*I degree*	**21**	**7**	2	7	*p* = 0.05
*II degree*	**15**	**15**	4	9	
*Wheezing*					RSV vs. no
*Mild*	**3**	**0**	0	**10**	*p* = 0.003
*Moderate*	**25**	**16**	5	**10**	RSV vs. no
*Severe*	8	6	1	4	*p* = 0.003
*Duration in hospital (days)*	**7 (6–8)**	**6 (5–7.25)**	6 (5–7)	6.5 (5.2–7)	RSV vs. RV *p* = 0.037
*Recurrence*					
*Yes*	17	12	0	12	
*No*	19	10	6	12	
*CRP*	7.93 ± 19.93	15.74 ± 26.8	7.25 ± 6.88	4.32 ± 4.66	*p* = 0.173
*Leukocytes*	11.05 ± 4.07	13.42 ± 3.95	9.42 ± 3.36	12.01 ± 3.71	*p* = 0.079

**Table 2:** Serum levels of IL-10 and IFN-γ in relation to the identified virus infection.

	IL-10 (pg/mL)		IFN-γ (pg/mL)	
	Median IQR (25–75%)	*p*-Value	Median IQR (25–75%)	*p*-Value
**Type of Virus**				
RSV	14.4 (12.2–24.0)		3.21 (1.58–6.78)	
RV	8.9 (7.2–12.5)	<0.001 *	2.85 (1.12–7.32)	0.781
Other viruses				
	6.65 (3.9–15.3)	0.003 *	6.1 (1.34–7.28)	0.496
Undetected viruses				
	9.1 (6.35–10.45)	<0.001 *	4.66 (1.57–8.68)	0.376
*p-Value within the group*	**<0.001** **		0.660	
** Kruskal–Wallis test				
* Mann–Whitney U-test				

**Table 3:** Total serum levels of IL-10 and IFN-γ concerning respiratory failure and wheezing according to virus type. The cytokine concentrations are presented as the median with interquartile range (IQR 25–75%). The results for CRP and number of leucocytes are presented as the mean ± SD.

*IL-10*	*RSV*	*RV*	*Others*	*Undetected Virus*	*Intergroup*
	*n = 36*	*n = 22*	*n = 6*	*n = 24*	*Significance*
*Respiratory failure*					
*No*	-	-	-	10.2 (6.3–10.4)	
*I degree*	14.4 (12.8–27.9)	9.9 (8.9–11.1)	11.1 (6.8–15.3)	8.2 (7.2–9.1)	*p* = 0.005
*II degree*	14.4 (10.3–18.4)	8.3 (7.1–14.8)	5.2 (3.6–13.4)	9.4 (6.1–13.2)	
*Wheezing*					
*Mild*	12.8 (12.4–15.6)	-	-	9.5 (6.6–10.3)	
*Moderate*	14.4 (12.6–27.9)	8.9 (7.4–11.1)	6.5 (3.9–6.8)	10.1 (5.0–13.2)	*p* = 0.017
*Severe*	14.1 (10.3–19.8)	9.8 (7.0–27.0)	20.20	7.5 (6.5–8.5)	
***IFN-γ***					
Respiratory failure					
*No*	-	-	-	4.7 (2.2–5.7)	
*I degree*	5.2 (1.6–10.4)	3.4 (1.2–5.8)	12.1 (5.5–18.7)	8.2 (2.0–13.7)	*p* = 0.441
*II degree*	2.5 (1.6–3.3)	2.8 (1.6–8.2)	4.05 ± 3.41	4.6 (1.0–8.6)	
Wheezing	5.4 (5.3–7.9)	-	-	4.8 (2.2–25.4)	
Mild	3.3 (1.5–8.6)	3.3 (1.9–8.0)	5.5 (1.3–6.7)	4.3 (1.0–6.0)	
Moderate Severe	2.0 (1.4–3.1)	1.5 (0.6–2.6)	7.28	8.7 (4.4–13.7)	*p* = 0.189
CRP	7.93 ± 19.93	15.74 ± 26.80	7.25 ± 6.88	4.32 ± 4.66	*p* = 0.173
Leucocytes	11.05 ± 4.07	13.42 ± 3.95	9.42 ± 3.36	12.01 ± 3.71	*p* = 0.079

**Table 4:** Correlation between the serum levels of cytokines and the clinical characteristics of bronchiolitis in Bulgarian children.

	IL-10	IFN-γ	Type of Virus	Respiratory Failure	Wheezing	Recurrence
IL-10	1.000	0.055	0.546 ** ***p* < 0.001**	0.055	0.098	0.002
IFN-γ	0.055	1.000	0.097	0.163	0.192	0.1
Type of virus	0.546 ** ***p* < 0.001**	0.097	1.000	0.110	0.219 * ***p* = 0.041**	0.021
Respiratory failure	0.055	0.163	0.110	1.000	0.523 ** ***p* < 0.001**	0.055
Wheezing	0.098	0.192	0.218	0.523 ** ***p* < 0.001**	1.000	0.350 ** ***p* < 0.001**
Recurrence	0.002	0.1	0.021	0.055	0.350 ** ***p* < 0.001**	1.000

Correlation coefficient: Spearman’s rho. * -weak and ** -strong correlation coefficient

## Data Availability

The data presented in this study are available upon request from the corresponding author.
